# Diet adaptation in dog reflects spread of prehistoric agriculture

**DOI:** 10.1038/hdy.2016.48

**Published:** 2016-07-13

**Authors:** M Arendt, K M Cairns, J W O Ballard, P Savolainen, E Axelsson

**Affiliations:** 1 Science for Life Laboratory, Department of Medical Biochemistry and Microbiology, Uppsala University, Uppsala, Sweden; 2 School of Biotechnology and Biomolecular Sciences, Faculty of Science, University of New South Wales, Sydney, NSW, Australia; 3 Science for Life Laboratory, School of Biotechnology, Royal Institute of Technology (KTH), Solna, Sweden

## Abstract

Adaptations allowing dogs to thrive on a diet rich in starch, including a significant *AMY2B* copy number gain, constituted a crucial step in the evolution of the dog from the wolf. It is however not clear whether this change was associated with the initial domestication, or represents a secondary shift related to the subsequent development of agriculture. Previous efforts to study this process were based on geographically limited data sets and low-resolution methods, and it is therefore not known to what extent the diet adaptations are universal among dogs and whether there are regional differences associated with alternative human subsistence strategies. Here we use droplet PCR to investigate worldwide *AMY2B* copy number diversity among indigenous as well as breed dogs and wolves to elucidate how a change in dog diet was associated with the domestication process and subsequent shifts in human subsistence. We find that *AMY2B* copy numbers are bimodally distributed with high copy numbers (median 2*n*_*AMY2B*_=11) in a majority of dogs but no, or few, duplications (median 2*n*_*AMY2B*_=3) in a small group of dogs originating mostly in Australia and the Arctic. We show that this pattern correlates geographically to the spread of prehistoric agriculture and conclude that the diet change may not have been associated with initial domestication but rather the subsequent development and spread of agriculture to most, but not all regions of the globe.

## Introduction

Despite considerable efforts to understand the timing and location of dog domestication much uncertainty still remains. The oldest confirmed dog remains date to between 12 500 and 16 000 years before present (YBP) in Europe (Nobis, 1979; Baales, 1996; Boudadi-Maligne and Escarguel, 2014; Morey, 2014). Domestication in Europe is consistent with close mitochondrial DNA relatedness between ancient and extant European wolves and modern dogs (Thalmann *et al.*, 2013), but patterns of genetic variation indicate a southern East Asian, Mongolian or Nepalese origin of dogs (Pang *et al.*, 2009; Shannon *et al.*, 2015; Wang *et al.*, 2015). Analyses of whole-genome sequence data place extant wolves as sister clade to modern dogs, with no leads on the location of dog domestication, but argue for a single event that started between 11 000 and 16 000 YBP (Freedman *et al.*, 2014). Molecular dating however hinges on accurate mutation rates, and calibration of molecular clocks using DNA from fossil remains dates the most recent common ancestor of main dog mitochondrial DNA clades to 15 100–24 000 YBP (Thalmann *et al.*, 2013) and pushes autosomal estimates of the dog and wolf divergence to between 27 000 and 40 000 YBP (Skoglund *et al.*, 2015). Dogs may thus have been domesticated somewhere in Eurasia 11 000–40 000 YBP.

Detailed characterizations of phenotypic adaptations accompanying the transition from wolf to dog may provide additional perspectives on the origin of dogs by illuminating the environmental and cultural context of the domestication process. Genome-wide comparisons of dogs and wolves have started to dissect the genetic basis of adaptations during dog domestication (Axelsson *et al.*, 2013; Wang *et al.*, 2013), and based on enrichment analyses of genes targeted by selection it was concluded that adaptations in dog mainly fall into two major categories. First, in line with expectations that behavioural changes were important, selection frequently affected nervous system development (*P*_FDR_=0.013) genes (Axelsson *et al.*, 2013). Second, selection also targeted many digestion (*P*_FDR_=0.008) and fatty acid metabolism (*P*_FDR_=0.031) genes, indicating that domestication may have been accompanied by a change in diet. It was specifically demonstrated that (i) selection had targeted a duplication affecting the gene coding for pancreatic amylase (*AMY2B*), the enzyme that breaks starch into maltose in the small intestine, resulting in an average sevenfold *AMY2B* copy number expansion that is estimated to be associated with a 5.4% increase in serum amylase activity for each extra copy (Axelsson *et al.*, 2013; Arendt *et al.*, 2014). It was also demonstrated that selection likely targeted genes controlling (ii) the subsequent conversion of maltose to glucose (*MGAM*) and (iii) the transport of glucose across the small intestinal membrane (*SGLT1*) arguing that an entire pathway responsible for starch digestion and glucose absorption was altered in dog. This indicates that adaptations allowing dogs to thrive on a diet rich in starch, relative to the protein-based wolf diet, constituted a crucial step during dog domestication (Axelsson *et al.*, 2013).

Increased starch consumption may have accompanied several episodes in human history. First, whereas native starch granules resist digestion by α-amylase, gelatinization through heating significantly increases digestibility (Carmody and Wrangham, 2009) arguing that the introduction of cooking removed a significant hurdle to energy extraction from starch. Humans have known how to control fire for at least 300 000–400 000 years, but the timing of widespread cooking remains uncertain (Hardy *et al.*, 2015). Second, local demographic pressure from increasing human population sizes and deteriorating climate conditions may have forced humans to shift focus from large-sized game to small, low-quality game and increased exploitation of plant foods during the last glacial maximum (19 000–26 000 YBP) (Flannery, 1969). Analyses of use ware traces and plant residues on grinding stones provide evidence of widespread use of plant material, including wild cereals and tubers, throughout several regions of the globe by this time (Liu *et al.*, 2013). This period also coincided with the first appearance of pottery in East Asia—a technological innovation that facilitated both cooking and storage of starch-rich foods (Wu *et al.*, 2012). Finally, the development of agriculture (10 000–12 000 YBP) and subsequent full-scale farming for subsistence undoubtedly led to a significant increase in starch consumption. Stable isotope analyses indicate that Northern Levantine diets may have been dominated by plant materials by 10 500 YBP (Losch *et al.*, 2006) and that millet became a staple food in Northern Chinese diets by 7000 YBP (Hu *et al.*, 2008). Remains of paddy field systems (Cohen, 2011) and significant quantities of rice (Fuller *et al.*, 2009) indicate that large-scale rice farming had been established in the Yangtze region by 6500 YBP. From China, the Levant and other independent origins of agriculture farming subsequently spread to large parts of Eurasia, Africa and the Americas in prehistoric times (Diamond and Bellwood, 2003).

Parallel to the *AMY2B* expansion in dogs, a marked copy number expansion of the gene coding for salivary amylase (*AMY1*) in humans (2*n*_*AMY1*_ copy number range in humans: 2–16) likely reflects an adaptation to increased starch consumption among humans (Perry *et al.*, 2007). The *AMY1* copy number variation (CNV) thereby offers an additional window on past human diets. Higher *AMY1* copy numbers in extant farmers than in hunter gatherers likely confirm a significant increase in starch consumption during the Neolithic revolution. However, the presence of multiple *AMY1* copies in three pre-Neolithic European hunter and gatherers (2*n*_*AMY1*_=5, 6 and 13) (Lazaridis *et al.*, 2014; Olalde *et al.*, 2014) indicates a relatively strong dependence on starch also before the development of agriculture. Similarly, a lack of copy number expansion in both Neanderthals and Denisovians (2*n*=2) suggests that the divergence of *Homo sapiens* and Neanderthals at 550 000–765 000 YBP marks an upper limit for the onset of the human diet change (Prufer *et al.*, 2014). Thus, although the human starch consumption undoubtedly increased significantly during the Neolithic revolution, both genetic and archaeological evidence may hence be compatible with an upper Palaeolithic context of the dog diet change too.

If the diet of dogs changed during the Neolithic revolution, it is unlikely to have been linked to initial domestication as this process probably started (11 000–40 000 YBP) before the development of agriculture (10 000–12 000 YBP) (Freedman *et al.*, 2014). Likewise, although an earlier diet change would be compatible with a pre-agricultural timing of dog domestication, a lack of *AMY2B* copy number expansion in Australian Dingoes and some Arctic dogs also suggest that the diet change may not have been associated with initial domestication but rather linked to a subsequent shift in human subsistence (Arendt *et al.*, 2014; Freedman *et al.*, 2014).

To increase our understanding of how the dog diet change relates to the domestication process and to the shifts in human subsistence, we perform the first global study of *AMY2B* CNV using accurate and reliable DNA quantification methods. Hereby we obtain the first detailed picture of the *AMY2B* copy number expansion allowing us to study the dog starch adaptation at a regional scale and in the light of alternative human subsistence strategies.

## Materials and methods

### Samples

To allow for a detailed analysis of the distribution of *AMY2B* copy numbers throughout the dog population we collected buccal swab or blood samples from 221 dogs, 95 of which represent native dogs from around the globe, including 25 Australian Dingoes, and 126 of which represent breed dogs. We also included 171 dogs (including 19 native dogs from Greenland) that were previously genotyped for *AMY2B* copy numbers (Arendt *et al.*, 2014) using the same method used in this study. In total 392 dogs representing 96 diverse breeds and native dogs were analysed in this study (Supplementary Table 1). To analyse the origin of the *AMY2B* copy number expansion we also analysed tissue or blood samples from 5 Bulgarian golden jackals, 1 coyote (from Minnesota) and a total of 51 wolves, from Africa (*n*=1), Croatia (9), Estonia (8), Israel (4), Sweden (1), Denmark (1) and the United States (12), and from 15 wolves from the Swedish zoo population, which originates from the natural wolf populations in the Baltic region (Sweden, Finland, Estonia, Latvia and Russia) (Supplementary Table 2).

### DNA extraction

DNA was extracted from EDTA blood using either manual salt extraction or the QIASymphony DNA Midi kit (Qiagen, Venlo, Netherlands) on the QIASymphony robot (Qiagen). DNA was extracted from muscle tissue using the DNeasy blood and tissue kit (Qiagen). Buccal swab samples were stored on FTA cards from which DNA subsequently was eluted using a boiling method as follows: first, 2–3 punches of each FTA card were placed in a tube and washed three times in 180 μl ddH_2_O; second, elution was done by adding 25 μl ddH_2_O to each sample and boiling this for 30 min at 95 °C.

### Copy number assay

We used droplet digital PCR (ddPCR) to quantify individual *AMY2B* copy numbers in all dogs except the Dingoes (see below). By allowing for an absolute measure of DNA molecules partitioned into thousands of droplets this method enables a precise estimation of DNA copy numbers (Hindson *et al.*, 2011; Pinheiro *et al.*, 2012). Compared with real-time PCR, ddPCR has been shown to reduce mean coefficients of variation by 37–86% and improve reproducibility by a factor of seven (Hindson *et al.*, 2013). DNA was digested with restriction enzyme DRAI (New England Biolabs, Ipswich, MA, USA) to separate individual amylase copies to allow for better partitioning, except for DNA eluted from FTA cards as the elution method (boiling) is expected to have sheared the DNA sufficiently. DRAI was selected for digestion as both the reference and target amplicons lack DRAI restriction sites and so are left intact. Probe and primers for the amylase target gene and the *c7orf28b* reference gene were designed as described in Axelsson *et al.* (2013). In addition, an alternative reference target, c7orf28b-3, that closely match the amylase target in size (amylase target size: 76 bp; c7orf28b target size: 91 bp; and c7orf28b-3 target size: 60 bp) was designed to exclude a potential amplification bias in heavily fragmented DNA. This reference target was used for DNA extracted from FTA cards. Primer and probe sequence for the c7orf28b primer and probes were as follows: c7orf28b-3, 5′-GGGAAACTCCACAAGCAATCA-3′ c7orf28b-3, 3′-GAGCCCATGGAGGAAATCATC-5′ c7orf28b_3, VIC-CACCTGCTAAACAGC. ddPCR was performed using the QX100 third-generation ddPCR system provided by BIO RAD (Hercules, CA, USA) (Hindson *et al.*, 2011) All FTA-eluted DNA samples were run in triplicate to compensate for potential biases from variable input DNA concentrations, and data were merged before copy number estimation. For Dingo samples digital PCR was carried out using the Fluidigm (San Francisco, CA, USA) BioMark HD microfluidics system on 48.770 assay chips, with eight wells per sample to ensure CNV resolution. Raw copy number data were rounded to the nearest whole number.

### Geographical origin of dogs

The origin of 114 individuals representing native dogs (referred to as ‘Native' in Supplementary Table 1) was set to sampling location. These dogs were collected from rural regions with small influx of foreign dogs arguing that extant dogs are likely to represent the original populations. Breed dogs were assigned geographical origin according to Federation Cynologique International (http://www.fci.be) (Supplementary Table 1) with the following exceptions: first, three breed dogs sampled in Japan (Kawakami-Dog, Ryukyu and Satsuma-Dog), one in Kirgizstan (Afghan hound) and one in Russia (Stepnaya) were not recognized by Federation Cynologique International but nevertheless assigned country of origin based on their well-established local status; second, American Staffordshire Terriers, Nova Scotia Duck-Tolling Retrievers and Australian Terriers were classified as European given their recent origin in societies founded by European settlers in North America and Australia, respectively.

### Analyses of regional CNV

We studied regional CNV among all dogs by grouping dogs into 10 large geographic areas, based on country of breed origin or sampling location. These regions were Europe (except Russia), Africa, South West Asia (Israel, Turkey, Iran, Syria and Kazakhstan), South Asia (India and Afghanistan), East Asia (China, Japan, Taiwan and Kyrgyzstan), Southeast Asia (Cambodia, Philippines, Indonesia, Thailand and New Guinea), Australia, Central America (Mexico and Peru), Arctic America (Alaska, Canada and Greenland) and Arctic Asia (Siberia in Russia). Association between *AMY2B* CNV and agriculture was tested by characterizing dogs as having either an agrarian or non-agrarian origin based on approximate estimates of the geographical spread of prehistoric agriculture (Diamond and Bellwood, 2003). As recent admixture, bottlenecks and strong artificial selection may have confounded the relationship between genetic signatures and geographical origin in some dogs—in particular among the breed dogs (Parker *et al.*, 2004)—we also reanalysed regional CNV in a subset of dogs that only included the 114 native dogs. We used Wilcoxon rank-sum tests to compare population-specific *AMY2B* copy numbers in both data sets.

## Results

### General *AMY2B* variation in wolf, golden jackal, coyote and dog

Diploid *AMY2B* copy numbers in 5 golden jackals and 1 coyote were fixed at two. Similarly, 49 out of 51 wolves carried two *AMY2B* copies, whereas a Danish wolf carried a single copy and an Israeli wolf three copies (Supplementary Table 2). This contrasts to a previous study that found >2 *AMY2B* copies in 16 out of 40 wolves and argues that the *AMY2B* duplication is rare in wolves. Overall, these results points to an ancestral copy number of 2*n*_*AMY2B*_=2 in canids.

**Figure 1:**
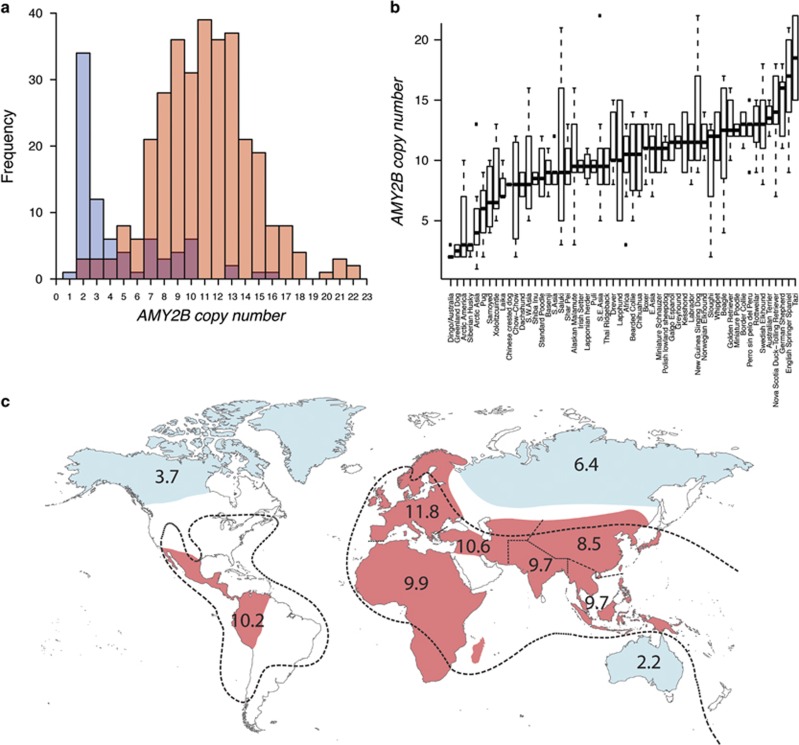
Worldwide *AMY2B* copy number distribution. (**a**) *AMY2B* copy numbers in 392 dogs are bimodally distributed with a major mode of 9 and a minor of 2. Red and blue colours depict dogs originating in agrarian and non-agrarian regions, respectively. Purple marks the overlap between agrarian and non-agrarian copy number distributions. (**b**) Box plot showing the *AMY2B* copy number distribution in 46 dog breeds and 114 native dogs grouped into 8 geographical regions (Africa, South West Asia (S.W. Asia), South Asia (S. Asia), East Asia (E. Asia), South East Asia (S.E. Asia), Australia, Arctic America and Arctic Asia). Horizontal bars display median copy number values and boxes represent the inter quartile range (IQR), with whiskers extending to maximum and minimum values within 1.5 × IQR. Black squares mark outliers. (**c**) Average *AMY2B* copy numbers in all dogs grouped in 10 large geographical regions: Europe, Africa, South West Asia, South Asia, East Asia, South East Asia, Australia, Central America, Arctic America and Arctic Asia. Dashed lines mark the approximate extension of prehistoric agriculture (Diamond and Bellwood, 2003) and colour marks regions that were sampled in this study and characterized as either agrarian (red) or non-agrarian (blue).

Among the 392 genotyped dogs diploid *AMY2B* copy numbers ranged from 1 to 22 with a median value of 2*n*_*AMY2B*_=10 (s.d.=4.3). The vast majority of dogs (*n*=354) carried more than 2 *AMY2B* copies with an overall fivefold copy number increase in dogs relative to wolves. However, rather than following a normal distribution as would be expected if dogs represent a homogenous population, copy numbers in dogs were bimodally distributed with a major mode of 9 and a minor of 2 (Figure 1a), indicating that the process underlying the *AMY2B* copy number expansion may have affected separate dog populations differently.

### Copy number distribution in dogs correlates with spread of prehistoric agriculture

Among the 37 dogs carrying the ancestral *AMY2B* copy number (2*n*_*AMY2B*_=2) 10 were indigenous sled dogs of Siberian, Canadian or Greenland origin, 1 was a Siberian husky, 1 was a Greenland dog and 22 represented Australian Dingoes (Supplementary Table 1); only 2 were of Chinese origin (one Chow-chow and one Pug) and a single dog, a Sloughi, originated in Africa. An indigenous sled dog from Chukotka, Siberia, carried only a single gene copy indicating that it was heterozygous for an *AMY2B* deletion. Similarly, among 46 dog breeds for which at least 2 individuals were genotyped, median *AMY2B* copy numbers was 3 or less in Greenland dog (median 2*n*_*AMY2B*_=2.5, Greenland) and Siberian husky (median 2*n*_*AMY2B*_=3, Siberia); and the 6 least-expanded breeds also included Samoyed (median 2*n*_*AMY2B*_=6.5, Siberia) and Laika (median 2*n*_*AMY2B*_=7, Siberia) (Figure 1b; Supplementary Table 3). Dogs from peripheral regions of the globe thus generally carried few *AMY2B* copies, although we note that high copy numbers in Alaskan Malamutes (median 2*n*_*AMY2B*_=9.5, Alaska) constitute a notable exception to this rule. Consistent with these observations, when all dogs are separated into 10 large geographical regions we observe overall low copy numbers in Australian (median 2*n*_*AMY2B*_=2, *n*=25, range: 2–3), Arctic American (median 2*n*_*AMY2B*_=3, *n*=37, range: 2–16) and Arctic Asian dogs (median 2*n*_*AMY2B*_=6, *n*=19, range: 1–13), relative to all other regions (Figure 1c).

We note that this copy number distribution generally follows a pattern that matches the approximate spread of prehistoric agriculture (Figure 1c). Arctic America, Arctic Asia and Australia represent regions with no or only recent agricultural practice, whereas prehistoric farming was widespread throughout most parts of the other areas surveyed here. To formally compare copy numbers in dogs from agrarian and non-agrarian regions we grouped dogs from Arctic America, Arctic Asia and Australia as non-agrarian regions and the remaining seven regions as agrarian regions. Copy numbers in 311 agrarian dogs were normally distributed with median 2*n*_*AMY2B*_=11 (range: 2–22, s.d.=3.5), which deviated significantly from the truncated normal distribution in 81 non-agrarian dogs with median 2*n*_*AMY2B*_=3 (range: 1–16, s.d.=3.5, *P*_Wilcoxon_<1 × 10^−16^; Figure 1a). Thus, by partitioning the data in agrarian and non-agrarian dogs the bimodal distribution originally observed across all dogs is resolved, indicating that historical presence and absence of agricultural practice separate dogs into two distinct populations with regards to *AMY2B* copy numbers.

### Native dogs

Recent admixture, bottlenecks and strong artificial selection have confounded the relationship between genetic signatures and geographical origin in some dogs (Parker *et al.*, 2004; Shannon *et al.*, 2015). These processes are likely to have affected breed dogs more than native dogs, and although significant European admixture has been documented in native dogs from the Southern Pacific and Latin America, regional ancestry components predominate in native dogs from remote areas of most other parts of the world (Shannon *et al.*, 2015). Thus, to limit the impact of recent demographic events and artificial selection on our analyses we analysed *AMY2B* CNV in 114 native dogs separately. We grouped these dogs into eight geographical regions (Australia, Arctic America, Arctic Asia, East Asia, South Asia, South East Asia, South West Asia and Africa) and, consistent with the analyses above, found that native dogs from Australia (median 2*n*_*AMY2B*_=2, *n*=25, range: 2–3), Arctic America (median 2*n*_*AMY2B*_=3, *n*=2, range: 2–10) and Arctic Asia (median 2*n*_*AMY2B*_=4, *n*=7, range: 1–13) carried markedly reduced *AMY2B* copy numbers relative to all other regions (Supplementary Figure 1; Supplementary Table 1). Overall, native dogs from regions that overlap with the geographical spread of prehistoric agriculture carried significantly more gene copies (median 2*n*_*AMY2B*_=10, *n*=60, range: 3–22, s.d.=3.0, *P*_Wilcoxon_<1 × 10^−15^) compared with dogs from the remaining three non-agrarian regions (that is, from Arctic America, Arctic Asia and Australia; median 2*n*_*AMY2B*_=2, *n*=54, range: 1–13, s.d.=2.4; Supplementary Figure 2). High copy numbers in the agrarian New Guinea Singing Dogs (median 2*n*_*AMY2B*_=9.5, *n*=4, range: 9–22) compared with the closely related non-agrarian Australian Dingoes (mean 2*n*_*AMY2B*_=2.1, *n*=25, range: 2–3) comprise a particularly striking example of this pattern.

### European dogs

It has been argued that some Nordic hunting and Nordic herding and watchdogs represent ancient breeds of Arctic European origin that hence may have evolved under limited influence of agriculture. Genetic data however indicate more recent shared ancestry with other European dogs for at least some of these breeds (Parker *et al.*, 2004; vonHoldt *et al.*, 2010). We genotyped 27 Nordic dogs, including Swedish and, Norwegian Elkhound, Norwegian Buhund, Norwegian Lundehund, Swedish Lapphund and Lapponian herders, and found overall high *AMY2B* copy numbers with a distribution that was indistinguishable (median 2*n*_*AMY2B*_=11, range: 5–18, s.d.=2.9) from that in other European dogs (median 2*n*_*AMY2B*_=12, *n*=174, range: 4–21, s.d.=3.1, *P*_Wilcoxon_=0.75).

## Discussion

In this study we identify a clear geographical pattern in the extent to which *AMY2B* copy numbers have expanded throughout the global dog population. We show that *AMY2B* copy numbers are bimodally distributed and that dogs originating in regions where agriculture was practiced in prehistoric times carry significantly more copies than dogs originating elsewhere. Our findings argue that the process underlying the adaptation to starch-rich foods has affected separate dog populations differently and that this difference may be ascribed to the development and spread of agriculture, or subsequent agro technological advances, to most, but not all regions of the globe.

### *AMY2B* copy numbers increased fivefold during dog domestication

Our analyses of a worldwide sample of dogs (2*n*_*AMY2B*_=10, range: 1–22, *n*=392) and the hitherto largest wolf sample examined (2*n*_*AMY2B*_=2, range: 2–3, *n*=51) show that *AMY2B* copy numbers underwent an average fivefold increase during dog domestication. This is a smaller increase and a narrower copy number range than previously reported (Axelsson *et al.*, 2013). These differences may reflect more precise DNA copy number estimations using ddPCR, a method that in particular has the potential to overcome the limited capacity of real-time quantitative PCR to resolve high copy number gene duplications accurately (Hindson *et al.*, 2011; Pinheiro *et al.*, 2012). Diploid copy numbers of two (2*n*_*AMY2B*_=2) in five golden jackals and a single coyote argue for an ancestral canid copy number of two. The initial duplication therefore likely represents a derived state in the lineage leading to dogs rather than being introgressed from any of these species. In contrast to a previous real-time quantitative PCR-based study that noted *AMY2B* duplications in 16 out of 40 wolves (Freedman *et al.*, 2014), our observation of two *AMY2B* copies in 49 out of 51 wolves argues that the duplication was rare in wolves. The discrepancy between these studies may be due to a difference in sample representation but may also reflect the lower resolution of real-time quantitative PCR compared with digital PCR. The scarcity of duplications in wolves observed here may indicate that selection for efficient starch digestion acted on a novel mutation in dogs, rather than on standing genetic variation in a common dog and wolf population, in particular considering evidence of admixture between dogs and Middle Eastern wolves (Freedman *et al.*, 2014).

The bimodal, rather than unimodal, copy number distribution in dogs, with a major mode of 9 and a minor of 2 (Figure 1), shows that the copy number expansion affected most but not all dog lineages and indicates that the process underlying the *AMY2B* expansion separates dogs into two distinct populations. Among dogs that lack duplications (2*n*_*AMY2B*_=2) we note that the majority (*n*=38) belong to dogs of either Arctic (*n*=13) or Australian (*n*=22) origin. Similarly, whereas we observe clearly expanded *AMY2B* arrays in 44 out of 46 breeds, we find few copies in Greenland dog and Siberian husky. This pattern is compatible with several possible scenarios for how the diet change may have been associated with the domestication process. It fits a scenario whereby dogs were first domesticated and spread across the globe followed by a subsequent diet change and copy number expansion throughout most of the dog population. Alternatively, if the diet change was associated with the onset of the domestication process, previously acquired *AMY2B* copies may later have become lost following a relaxation of the selection pressure as dogs dispersed to regions (such as the Arctic and Australia), where they hypothetically reverted to a carnivorous diet. This scenario would however imply independent copy number loss through drift in two diverse dog populations, unless the additional *AMY2B* copies exerted a significant fitness cost. While it is also possible that Greenland dogs and Siberian Huskies may have undergone secondary copy number loss through introgression from wolves after initial dog domestication (Skoglund *et al.*, 2015), this is unlikely to explain the lack of *AMY2B* expansion (2*n*_*AMY2B*_=2) in Australian Dingoes considering the absence of wolves in Australia. Finally, it should also be noted that these inferences may be confounded by the drastic founder effect(s) associated with the introduction of Dingoes to Australia and a lack of a full understanding of the role of Dingoes during the early phase of dog domestication.

### *AMY2B* copy number expansion correlates with spread of prehistoric agriculture

High copy numbers in central regions of the globe relative to in Australia, Arctic Asia and Arctic America form a pattern that broadly matches the approximate maximum spread of prehistoric agriculture (Diamond and Bellwood, 2003) (Figure 1c). Overall, we find that dogs from regions with prehistoric agriculture carry significantly more *AMY2B* copies (median 2*n*_*AMY2B*_=11, range: 2–22, *n*=311) than dogs from regions outside of this area (median 2*n*_*AMY2B*_=3, range: 1–16, *n*=81) and that the bimodal copy number distribution observed in the global dog population is nearly completely resolved by partitioning dogs into those originating in regions with and without prehistoric agriculture, respectively (Figure 1a). These observations argue that the diet adaptations affected separate dog populations differently and that this difference may be ascribed to a drastic diet shift associated with either the development and spread of agriculture or subsequent refinements of cultivation techniques to most, but not all, regions of the globe. More specifically, the lack of *AMY2B* expansions in Dingoes suggests that the dog diet adaptation may have started after the arrival of Dingoes in Australia between 5000 and 3500 YBP. This conclusion is however contradicted by significant copy number gains in the closely related New Guinea Singing dog and would necessitate that New Guinea Singing dogs acquired additional *AMY2B* copies through introgression from dogs entering South East Asia at a later stage. Alternatively, if the diet adaptation started earlier and Australian Dingoes indeed originated from a population that carried extra *AMY2B* copies these could potentially have been lost when Dingoes reverted to an ecological niche similar to that of wolves or as a result of the strong founder effects associated with the transfer to Australia. That is, whereas the geographical *AMY2B* copy number pattern observed here is consistent with a diet change associated with agricultural practice, a more precise timing of this event remains to be determined.

Recent admixture, bottlenecks and strong artificial selection are likely to have confounded the relationship between genetic signatures and geographical origin in some of the dogs analysed here (Parker *et al.*, 2004; Shannon *et al.*, 2015). For instance, we note that 8 out of 10 Alaskan Malamutes carry 9 or more *AMY2B* copies (median 2*n*_*AMY2B*_=9.5, range: 3–16), which clearly deviates from the low copy number distribution observed in other dogs assumed to have originated in the Arctic (Figure 1a). This pattern is however compatible with large-scale analyses of genetic variation that indicates recent European introgression into the Alaskan Malamute breed (van Asch *et al.*, 2013; Shannon *et al.*, 2015). Similarly, we also note that Central American breeds analysed in this study, including Chihuahua, Perro sin pelo del Peru and Xoloitzcuintle, are likely to have experienced significant recent introgression from European dogs arguing that our results may not reflect pre-Colombian *AMY2B* copy number patterns accurately (Shannon *et al.*, 2015). Along the same lines, indistinguishable copy number distributions in Nordic hunting and Nordic herding and watch dogs (median 2*n*_*AMY2B*_=11, *n*=27, range: 5–18, s.d.=2.9) relative to other European dogs (median 2*n*_*AMY2B*_=12, *n*=174, range: 4–21, s.d.=3.1) may indicate introgression from agrarian dogs or a relatively recent shared origin with other European dogs, rather than a separate and older origin of these dogs in Arctic Europe (Parker *et al.*, 2004; vonHoldt *et al.*, 2010).

In contrast to breed dogs, high effective population sizes of native dogs argue that they are less affected by bottlenecks and artificial selection, and therefore should reflect the ancient genetic structure of dogs more accurately if recent admixture has been limited (Shannon *et al.*, 2015). Native dogs analysed here were sampled in rural areas of geographical regions in which the regional ancestry components generally predominate (Shannon *et al.*, 2015) arguing that admixture had a limited effect on the *AMY2B* CNV in these dogs. We note that our separate analyses of native dogs recapitulates the geographical copy number pattern observed among all dogs; that is (i) native dogs that lack the *AMY2B* expansion (2*n*_*AMY2B*_=2) generally originate in Arctic regions or Australia and (ii) native dogs from regions with prehistoric agriculture carry significantly more copies than those originating elsewhere (Supplementary Figure 1). The general patterns observed in this study are therefore likely to be relatively robust to recent admixture.

We conclude that significantly elevated *AMY2B* copy numbers in dogs from regions with prehistoric agriculture indicate that the dog diet change is likely to be associated with the Neolithic, but note that it is premature to entirely exclude both an earlier onset of this change given evidence of increased human starch consumption before this episode, as well as a later onset based on the lack of *AMY2B* duplications in Dingo. Given a Neolithic association of the diet change and the fact that current estimates of the timing of dog domestication predates the development of agriculture, a widespread lack of copy number expansions in Arctic and Australian dogs is consistent with onset of selection for efficient starch digestion after initial domestication, although we acknowledge that founder effects, relaxed selection, introgression from wolves and the role of Dingoes during the domestication process could have confounded these interpretations. Future analyses of *AMY2B* copy number patterns in fossil dog remains are likely to provide additional detail with regards to the precise timing of this selection event.

## Data Archiving

Data available from the Dryad Digital Repository: http://dx.doi.org/10.5061/dryad.1j6b6.
