# Supplementary figures and images for: Diet adaptation in dog reflects spread of prehistoric agriculture
Source: Heredity (Edinb). 2016 Jul 13;117(5):301–6. doi: 10.1038/hdy.2016.48 (PMC5061917; doi:10.1038/hdy.2016.48)

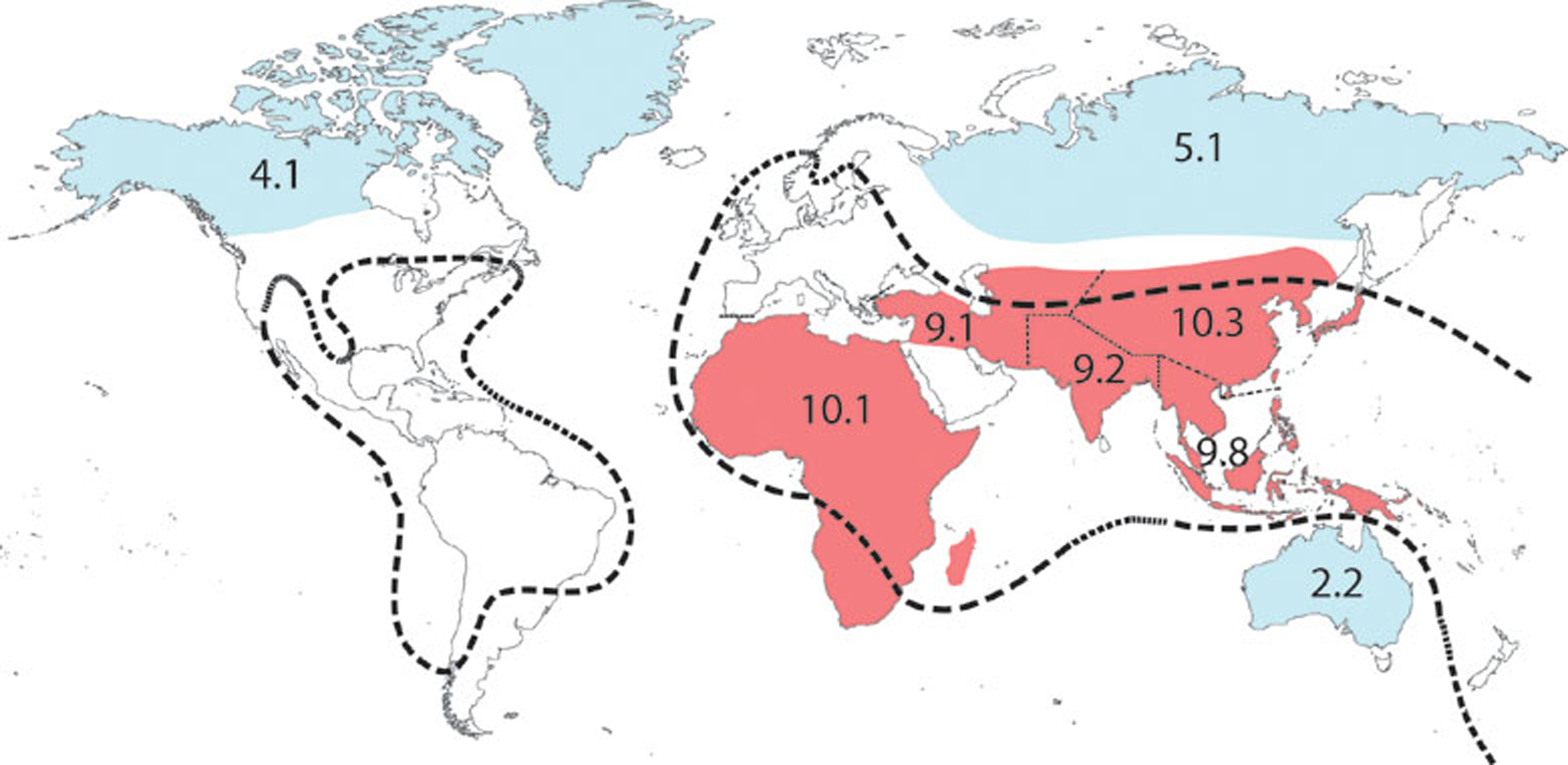

Supplement: Supplementary Figure 1 [file hdy201648x1.tif]

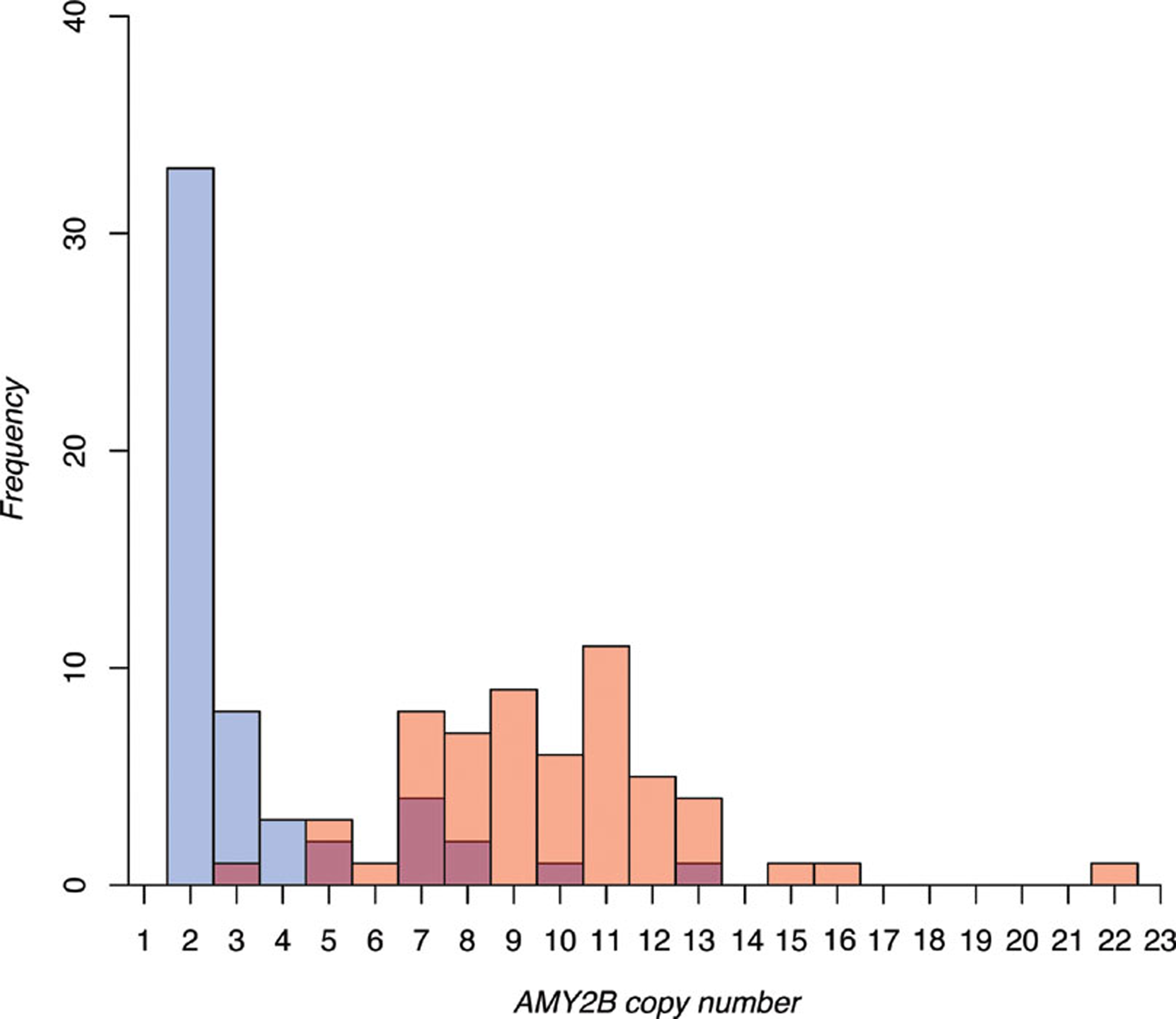

Supplement: Supplementary Figure 2 [file hdy201648x2.tif]
