# Supplementary material for: Diet adaptation in dog reflects spread of prehistoric agriculture
Source: Heredity (Edinb). 2016 Jul 13;117(5):301–6. doi: 10.1038/hdy.2016.48 (PMC5061917; doi:10.1038/hdy.2016.48)
Supplement: Supplementary Legends [file hdy201648x6.docx]

**Supplementary material.**

**Supplementary table 1. Summary of all dogs analysed in this study.** *“AMY2B* copy numbers”: Rounded *AMY2B* copy number of individual; “Name”: name of dog lineage (breed name or name of native dog population if applicable); “Native/Breed”: states whether the individual was analysed as a breed or a native dog; “Country of origin” and “Region of origin”: see Methods section for information on how geographical origin was set,: “Culture”: set to agrarian or non-agrarian depending on whether region of origin overlaps with the approximate spread of pre-historic agriculture.

**Supplementary table 2. Summary of other canids analysed in this study.** *“AMY2B* copy numbers”: Rounded *AMY2B* copy number of individual.

**Supplemlentary table 3. Summary statistics of *AMY2B* copy number distribution in dogs.** *AMY2B* copy number distribution in breeds for which at least two individuals were genotyped and in all native dogs grouped into 8 geographical regions (Africa, South West Asia (S. W. Asia), South Asia (S. Asia), East Asia (E. Asia), South East Asia (S. E. Asia), Australia, Arctic America and Arctic Asia). “min” and “max” shows the minimum and maximum copy number observed through out “n” dogs analysed. “sd” shows standard deviation.

**Supplementary Figure 1. World map of *AMY2B* copy number distribution in native dogs.** Average *AMY2B* copy numbers in 114 native dogs grouped in 8 large geographical regions: Africa, South West Asia, South Asia, East Asia, South East Asia, Australia, Arctic America and Arctic Asia. Dashed lines mark the approximate extension of pre-historic agriculture (Diamond and Bellwood, 2003) and colour marks regions that were sampled in this study and characterised as either agrarian (red) or non-agrarian (blue).

**Supplementary Figure 2. *AMY2B* copy number distribution in native dogs.** *AMY2B* copy numbers in 114 native dogs are bimodally distributed with a major mode of 9.5 and a minor of 2. Red and blue colours depict dogs originating in agrarian and non-agrarian regions, respectively. Purple marks the overlap between agrarian and non-agrarian copy number distributions.
